# Prediction of early recurrence of pancreatic ductal adenocarcinoma after resection

**DOI:** 10.1371/journal.pone.0249885

**Published:** 2021-04-12

**Authors:** Toshitaka Sugawara, Daisuke Ban, Jo Nishino, Shuichi Watanabe, Aya Maekawa, Yoshiya Ishikawa, Keiichi Akahoshi, Kosuke Ogawa, Hiroaki Ono, Atsushi Kudo, Shinji Tanaka, Minoru Tanabe

**Affiliations:** 1 Department of Hepatobiliary and Pancreatic Surgery, Graduate School of Medicine, Tokyo Medical and Dental University, Tokyo, Japan; 2 Division of Bioinformatics, Research Institute, National Cancer Center Japan, Tokyo, Japan; 3 Department of Molecular Oncology, Graduate School of Medicine, Tokyo Medical and Dental University, Tokyo, Japan; Centro Nacional de Investigaciones Oncologicas, SPAIN

## Abstract

**Background:**

Even after curative resection, pancreatic ductal adenocarcinoma (PDAC) patients suffer a high rate of recurrence. There is an unmet need to predict which patients will experience early recurrence after resection in order to adjust treatment strategies.

**Methods:**

Data of patients with resectable PDAC undergoing surgical resection between January 2005 and September 2018 were reviewed to stratify for early recurrence defined as occurring within 6 months of resection. Preoperative data including demographics, tumor markers, blood immune-inflammatory factors and clinicopathological data were examined. We employed Elastic Net, a sparse modeling method, to construct models predicting early recurrence using these multiple preoperative factors. As a result, seven preoperative factors were selected: age, duke pancreatic monoclonal antigen type 2 value, neutrophil:lymphocyte ratio, systemic immune-inflammation index, tumor size, lymph node metastasis and is peripancreatic invasion. Repeated 10-fold cross-validations were performed, and area under the receiver operating characteristic curve (AUC) and decision curve analysis were used to evaluate the usefulness of the models.

**Results:**

A total of 136 patients was included in the final analysis, of which 35 (34%) experienced early recurrence. Using Elastic Net, we found that 7 of 14 preoperative factors were useful for the predictive model. The mean AUC of all models constructed in the repeated validation was superior to the standard marker CA 19–9 (0.718 vs 0.657), whereas the AUC of the model constructed from the entire patient cohort was 0.767. Decision curve analysis showed that the models had a higher mean net benefit across the majority of the range of reasonable threshold probabilities.

**Conclusion:**

A model using multiple preoperative factors can improve prediction of early resectable PDAC recurrence.

## Introduction

Pancreatic ductal adenocarcinoma (PDAC) is a devastating disease with a 5-year survival rate of only approximately 10% [1]. Despite current multimodality screening methods, many patients suffer from locally advanced or metastatic disease at the time of initial presentation, which results in up to 80% being diagnosed as unresectable [2]. In the minority of patients able to undergo surgical resection with curative intent, the standard treatment for improved survival is adjuvant chemotherapy. To the present, several studies have reported the role and regimens of adjuvant chemotherapies, from single-agent therapies to combination chemotherapies [3–7]. However, even in patients receiving adjuvant chemotherapy, early systemic recurrence (ER) is a major problem for as many as half of them [8–10]. This may be because these patients already have micrometastases at the time of surgery, preventing them from achieving durable remission and long survival [11, 12]. It is possible that because such patients will not receive much benefit from surgery, alternative treatment options would be a better option. Hence, it is important to be able to accurately predict which patients are most likely to suffer ER and to consider alternative treatments for them. Indeed, several studies have attempted to discover markers allowing accurate prediction of ER after resection of PDAC [9, 13–17]. In this way, some single risk factors have been identified (e.g. CA 19–9, tumor size, etc.), as well as some scoring methods combining several different preoperative factors. However, to be clinically useful, risk must be quantified rather than merely identified as present or not present, as has thus far been the case with single factor predictors, or multifactorial scoring models with low accuracy. More accurate predictions can and must be made by modeling outcomes using multiple appropriately selected factors in combination. Hence, in the present study, we sought to construct a model using the most relevant constellations of preoperative parameters to accurately predict early recurrence after PDAC resection.

## Patients and methods

Data on 184 patients with PDAC who underwent pancreatectomy in Tokyo Medical and Dental University, Medical Hospital between January 2005 and September 2018 were retrospectively reviewed. Patients with borderline resectable PDAC, and patients who received neoadjuvant therapy or total pancreatectomy for recurrence in the remnant pancreas were excluded, as were all patients with postoperative follow-up of <6 months. Patients whose CA 19–9 levels were persistently < 5 were deemed likely to be Lewis antigen negative and were also excluded. This resulted in a study cohort of 136 patients with resectable PDAC. After the final predictive model was constructed, additional 15 patients who underwent surgery between November 2018 and December 2019 were included in the validation cohort. The cut-off for early recurrence was defined as 6 months in consideration of the time required for micrometastases to become detectable.

This study was approved by the Medical Ethical Committee in Tokyo Medical and Dental University, Medical Hospital (No.: M2000-1080), and all patients provided informed consent to have data from their medical records used for research purposes preoperatively. The patients’ unanonymized medical records were accessed for 2 months.

Preoperative data on demographics, tumor markers, blood immune-inflammatory factors (neutrophil:lymphocyte ratio (NLR), platelet:lymphocyte ratio (PLR), total lymphocyte count (TLC), C-reactive protein (CRP):albumin ratio (CAR), modified Glasgow prognostic score (mGPS) [18] and systemic immune-inflammation index (SIII) [16], as well as clinicopathological data were analyzed. PDAC stage classification was based on the 8th edition of the UICC TNM classification. The clinical peripancreatic invasion (cPI), clinical T stage (cT), and lymph node metastasis (cLNM) were measured using preoperative contrast-enhanced abdominoperineal computed tomography. The presence of lymph nodes larger than 10 mm in diameter was considered as cLNM. The definition of cPI was a tumor extended to the surface of the pancreas and peripancreatic fat tissue. Potential curability of the resections was coded as R0 (no residual tumor) or R1 (microscopic residual tumor). Immune-inflammatory factors were defined as absolute neutrophil count divided by absolute lymphocyte count (NLR), absolute platelet count divided by absolute lymphocyte count (PLR), CRP divided by albumin (CAR), modified Glasgow prognostic score (mGPS scale 0–2, one point each for CRP >0.5 mg/dL or albumin <3.5 g/ dL), and SIII (platelet count multiplied by NLR).

The software packages SPSS® version 20 (IBM, Armonk, New York, USA) and R version 3.6.1 (http://www.r-project.org) were used for statistical analysis. Continuous data are reported as medians (interquartile range [IQR]). Categorical variables are reported as frequency (n) and percentage (%). For continuous variables, comparisons were made using the Mann-Whitney U test. For categorical variables, comparisons were made using Chi square or Fisher’s exact tests. Uni- and multivariate logistic regression analyses were performed to identify factors predictive of early recurrence. The Kaplan–Meier method was used to estimate median relapse-free survival (RFS) and overall survival (OS). Survival was determined as the time from the day of surgical resection. Before their use for constructing the models, continuous variables were logarithmically transformed and all missing values were imputed by the machine learning method “factor analysis of mixed data” using the missMDA package in R.

## Model construction

Least absolute shrinkage and selection operator (LASSO) regression is a well-recognized method for sparse modeling and for the selection from high dimensional data of the most important variables influencing a designated outcome, thus providing interpretable models [19]. However, when a set of variables is correlated, LASSO tends to select only one of them. In the present study, some preoperative features independently associated with the outcome of interest could well be highly correlated with one another and therefore missed by LASSO. While Ridge regression can also include such correlated factors, it retains all of them, and this can result in difficulties in interpreting the model [20]. For these reasons, we used Elastic Net, a fusion of LASSO and Ridge regression methodologies. which retains the strong points of both approaches while addressing the weak points of each [21].

Consider the data where *Y_i_* and *x_i_* = (*x*_*i*1_,…,*x_ih_*)^*t*^, which are binary outcomes (coded 1 for “early recurrence” and 0 otherwise) and a set of *h* preoperative features of *i*-th subject (*i* = 1,…,*n*), respectively. Let *l*(*β*; *Y_i_,x_i_,i* = 1,…,*n*) be the logistic log-likelihood, where *β* = (*β*_1_,…,*β_h_*)^*t*^ denotes the vector of regression coefficients. The Elastic Net estimates of *β* are the maximizers of

$$l(\beta ;Y_{i},x_{i},i=1,\ldots ,n)-\lambda P_{\alpha }(\beta ),$$

where $P_{\alpha }(\beta )=\sum_{j=1}^{h}\left(\frac{\left(1+\alpha \right)}{2}\beta_{j}^{2}+\alpha |\beta_{j}|\right)$ and λ ≥ 0 is the complexity parameter, and 0 ≤ α ≤ 1 is the compromise between ridge (α = 0) and LASSO (α = 1). We used the Caret package in R to construct a model using Elastic Net.

## Evaluation of predictive ability

To evaluate the predictive ability of the model constructed by Elastic Net from preoperative data, we adopted 10-fold cross validation [22], repeated 1000 times to compensate for lack of external validation. For each cross-validation iteration, a predictive model was constructed using only the training samples, and validation (the evaluation of predictive ability) of the model was achieved using the test samples independent of the training samples. To determine the best value for α and λ given a training set, a grid search was performed to accomplish a cross-validation within it, i.e., according to the “nested” cross-validation procedure, and a model was constructed. Nested cross-validation is a commonly used approach to model selection with hyperparameter optimization which avoid optimistically-biased estimates of model predictive performance [23]. To evaluate the usefulness of the model, area under the curve (AUC) and decision curve analyses were applied. The latter is a method to evaluate the clinical consequences of model predictions by comparing net benefit, calculated by summing the benefits (true-positive rate) and subtracting the harms (false-positive rate) [24]. Decision curves do not represent the likelihood of survival, but rather help determine which models should be used in specific clinical situations represented by a given probability threshold. This helps clinicians avoid unnecessary treatment.

Finally, after 1000 iterations of the 10-fold cross-validation, the average of the AUC and decision curve was taken. For comparison, the same validation was carried out for CA 19–9 (a conventional risk factor), as well as for the significant risk factors identified by multivariate analysis in this study, and also the score derived from a combination of CA 19–9 and PLR reported previously [17].

## Final predictive model

We developed a final model from all included patients, using Elastic Net. The scores for each patient (i.e., the linear combination of selected factors) were calculated from the final model. We then used X-tile software (version 3.6.1; Yale University School of Medicine, New Haven, Conn) [25], to determine an optimum cut-off value for the score most clearly stratifying low- and high- score patients into those with better or worse survival outcomes by the minimal *P*-value approach. To correct for over-fit bias, we utilized bootstrap methodology with 1000 resamples to calculate an optimism-corrected AUC [26, 27]. Bootstrapping methodology randomly repeats sampling from a population by replacing samples in the original dataset, and developing models for each sample. Applying each fitted model to the original sample and the bootstrapped sample, AUCs are calculated. The difference between the two AUCs is defined as the “optimism”. This process is repeated 1000 times and the optimism errors are averaged. The corrected accuracy of a model is defined as the result of subtracting optimism from the AUC of the final model. Bootstrapping methodology is often employed to validate a prediction model when a validation cohort is not available. Here, the performance of the model was also investigated by decision curve analysis and compared in the same way as with the 10-fold cross-validation.

## Results

**Fig 1:**
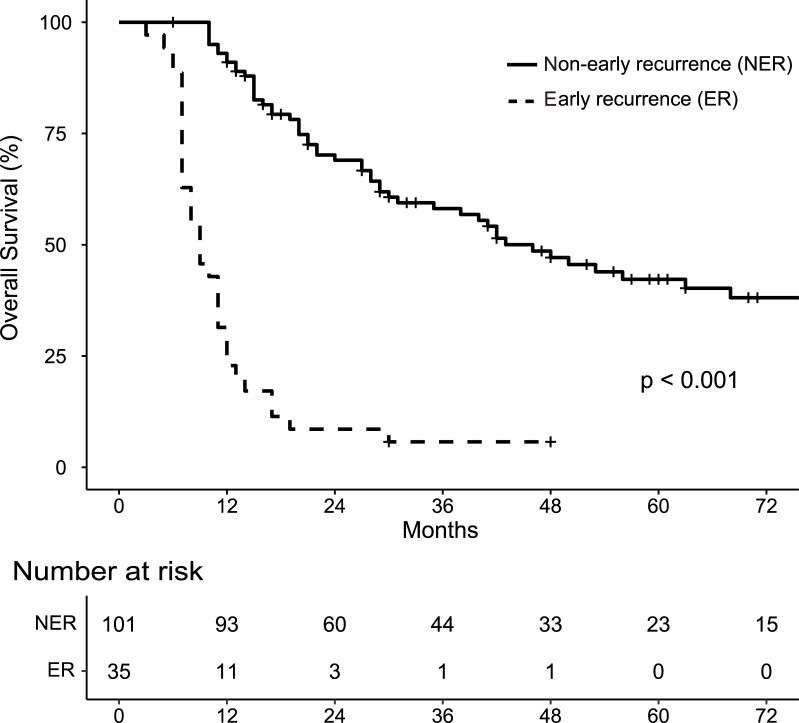
Kaplan-Meier analysis of overall survival.

A total of 136 patients with resectable PDAC was included in the final analysis. At the time of last follow-up, 101 patients (74.3%) had suffered recurrence, of which 35 (25.7%) had recurred within 6 months of surgery (designated the “early recurrence group”, ER group). Overall survival for patients in or not in the ER group is shown in Fig 1. The ER group as a whole had significantly worse OS with a median survival time of 9 months vs 46 months, p < 0.001. The preoperative clinical characteristics of the patients in the two groups are summarized in Table 1. The percentage of patients in the ER group with clinical peripancreatic invasion (cPI, p = 0.002) and lymph node metastasis (cLNM, p < 0.001) was higher than in the group without ER. Clinical tumor size was larger (cTS, p = 0.008) and the CA 19–9 level tended to be higher in the ER group (Table 1).

**Table 1:** Baseline clinicopathological characteristics of patients who did or did not suffer early recurrence.

	early recurrence (n = 35) (%)	no early recurrence (n = 101) (%)	p
Age (years)	72 (64–77)	67.5 (59–75)	0.156
Sex			1.000
Male	21 (60)	61 (60.4)	
Female	14 (40)	40 (39.6)	
Performance Status			0.516
0	27 (77.1)	70 (69.3)	
1	8 (22.9)	31 (30.7)	
Tumor location			0.645
Head	21 (60)	65 (64.4)	
Body and tail	14 (40)	36 (35.6)	
CEA (ng/mL)	3.25 (2.2–5.5)	3.0 (1.7–5.3)	0.479
CA 19–9 (U/mL)	193.8 (80.25–634.3)	63.3 (22.3–221.0)	0.051
DUPAN2 (U/mL)	140.0 (53.5–485.0)	64.5 (25.0–270.0)	0.078
Total bilirubin (mg/dL)	0.80 (0.60–1.35)	0.80 (0.60–1.70)	0.814
Direct bilirubin (mg/dL)	0.10 (0.10–0.25)	0.10 (0.10–0.20)	0.640
NLR	2.285 (1.735–2.975)	2.49 (1.965–3.285)	0.183
TLC	1309.5 (1026.5–1771)	1333 (1101–1622)	0.888
PLR	173.45 (110.9–207.75)	175.8 (125.55–239.2)	0.366
CAR	0.0375 (0.024–0.168)	0.028 (0.0115–0.0625)	0.555
SII	522.45 (323.85–753.1)	582.4 (400.95–856.85)	0.188
mGPS			0.832
0	3 (8.6)	6 (5.9)	
1	6 (17.1)	20 (19.8)	
2	26 (74.3)	75 (74.3)	
Clinical T stage^a^			0.008
1	6 (20.7)	44 (50.6)	
2	21 (72.4)	42 (48.3)	
3	2 (6.9)	1 (1.1)	
Clinical peripancreatic invasion^b^	29 (100)	66 (75.9)	0.002
Clinical lymph node metastasis^b^	11 (37.9)	6 (6.9)	< 0.001

^a^ UICC TNM classification

^b^ Binary variables.

NLR, neutrophil:lymphocyte ratio; TLC, total lymphocyte count

PLR, platelet:lymphocyte rate; CAR, C-reactive protein:albumin ratio

SII, systemic immune-inflammation index; mGPS, modified Glasgow prognostic score.

**Fig 2:**
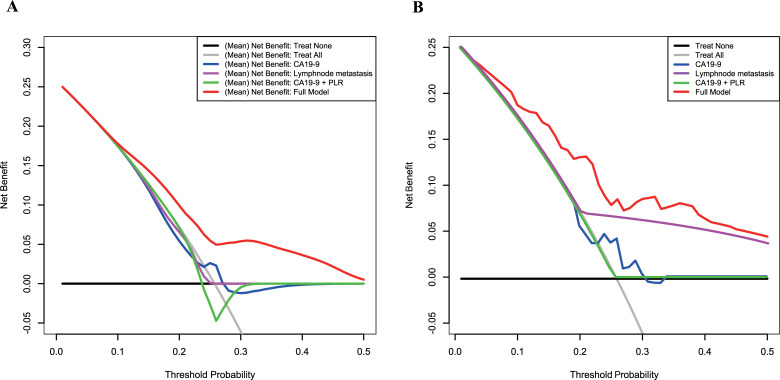
Decision curve analysis for the constructed models and other risk factors in (A) repeated 10-fold cross validation and (B) the entire cohort. The net benefit (y-axis) was calculated by summing the benefits (true-positive results) and subtracting the harms (false-positive results). The constructed models had the highest (mean) net benefit compared with the other assessments.

Factors identified as significantly different between the two groups by univariate analysis were then subjected to multivariate Cox regression analysis (Table 2). In this assessment, the only factor predicting ER was clinical lymph node metastasis (cLNM). Going further, we used all preoperative parameters listed in Table 1 for Elastic Net model construction, except for total and direct bilirubin (in order to exclude any influence of obstructive jaundice and biliary drainage). Additionally, in this cohort, the risk factors CA 19–9, cLNM, and a score derived from combining CA 19–9 and PLR (previously-reported prognostic factors) were also analyzed in comparison with our Elastic Net modeling. S1 Table shows the final input file for Elastic Net model construction. This revealed that the average AUC of all models in the repeated 10-fold cross-validations was better than the others tested (0.718), whereas average AUCs for CA 19–9, cLNM, and the score from CA 19–9 and PLR were 0.657, 0.643, and 0.621, respectively. Decision curve analysis showed that, on average, the models constructed by Elastic Net had a higher overall net benefit than any of the other approaches across most of the range of reasonable threshold probabilities (Fig 2A).

**Table 2:** Multivariate analysis of patients’ preoperative variables.

Variables	Hazard ratio (95%CI)	p
CA 19–9 (U/mL)	0.874 (0.524–1.46)	0.604
Clinical T stage^a^	1.75 (0.744–4.36)	0.212
Clinical peripancreatic invasion^b^	2.81×10^7^ (5.69×10^−24^–5.28×10^178^)	0.990
Clinical lymph node metastasis^b^	5.57 (1.88–18.1)	0.003

^a^ UICC TNM classification

^b^ Binary variables.

**Fig 3:**
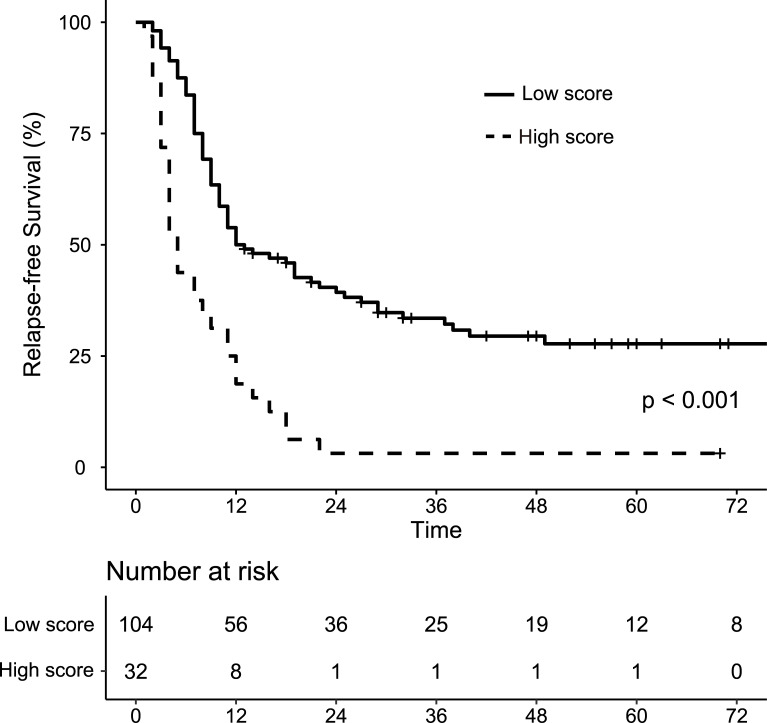
Kaplan-Meier analysis of relapse-free survival of patients with a high (≥ -0.90) versus low (< -0.90) Elastic Net model score in the whole cohort.

The model constructed from all included patients can be expressed as follows.

$$\begin{array}{l} logit(p)=log(\frac{p}{1-p})\\ \;=-1.45+0.00661\times Age(year)+0.101\times {log}_{10}DUPAN2(\frac{U}{mL})\\ \;-0.284\times {log}_{10}NLR-0.530\times {log}_{10}SIII+0.280\times cTS\\ \;+1.085\times cLNM+0.738\times cPI, \end{array}$$

where *p* is the probability of early recurrence, cTS is clinical tumor size (category value based on the 8^th^ edition of UICC TNM classification), cLNM is the presence of clinical lymph node metastasis and cPI is the presence of clinical peripancreatic invasion. The AUC of the model was 0.767. Bootstrapping analysis (i.e., resampling the model 1000 times) revealed a mean over-optimism value of 0.00192 and a corrected AUC of 0.765. Fig 2B shows the result of the decision curve analysis indicating that the model constructed by Elastic Net had the highest overall net benefit across the majority of the range of reasonable threshold probabilities. From X-Tile analysis taking relapse-free survival (RFS) as the outcome, the cutoff value of the model was determined as -0.90 (maximum high/low x^2^ = 28.24, Monte Carlo p <0.001, HR = 1.43). Fig 3 shows the RFS for patients of the high and low score groups stratified by this cutoff value, indicating that the RFS of the high score group was significantly poorer (median survival of 5 months vs 12.5 months, *p* <0.001). The predictive power of the final model was good (AUC = 0.867) in the validation cohort, which included 5 patients (33%) who suffered from early recurrence. Table 3 reveals the preoperative clinical characteristics of the patients in the validation group.

**Table 3:** Baseline clinicopathological characteristics of the validation cohort.

	validation group (n = 15) (%)
Age (years)	72 (70–78)
Sex	
Male	8 (53.3)
Female	7 (46.7)
CEA (ng/mL)	3.5 (2.1–5.0)
CA 19–9 (U/mL)	86.4 (40–268)
DUPAN2 (U/mL)	98.0 (55.5–290.0)
Total bililubin (mg/dL)	0.75 (0.70–1.05)
Direct bililubin (mg/dL)	0.10 (0.10–0.10)
NLR	3.962 (1.734–4.737)
TLC	855 (1127–1545.6)
PLR	174.21 (135.87–246.94)
CAR	0.0257 (0.00731–0.111)
SII	732.56 (399.78–1152.44)
mGPS	
0	11 (73.3)
1	4 (26.7)
2	0 (0)
clinical T satage^a^	
1	7 (46.7)
2	8 (53.3)
3	0 (0)
clinical peripancreatic invasion^b^	14 (93.3)
clinical lymph node metastasis^b^	3 (20.0)
Residual tumor	
R0	15 (100)
R1	0 (0)
Adjuvant chemotherapy	
Yes	11 (73.3)
No	4 (26.7)
Early recurrence	5 (33.3)

^a^ UICC TNM classification

^b^ Binary variables.

NLR, neutrophil to lymphocyte ratio; TLC, total lymphocyte count

PLR, platelet to lymphocyte rate; CAR, C-reactive protein to albumin ratio

SII, systemic immune-inflammation index

mGPS, modified grasgow modified glasgow prognostic score.

The pathological findings, the details of adjuvant therapy, and postoperative complications are shown in Table 4. The pathological tumor grade was significantly higher in the ER group as well as the clinical staging. The concordance rate between preoperative diagnosis (cTS, cLNM) and pathological diagnosis (pTS, pLNM) is 52.7% and 56.4% respectively, which is consistent with the previous reports [28, 29]. The proportion of patients who received adjuvant therapy and R0 resection, which may affect the outcomes, was not different between the two groups. None of the patients received adjuvant radiotherapy. In contrast, no ER had a higher incidence of grade III or higher surgical complications in the Clavien-Dindo classification and clinically relevant postoperative pancreatic fistulas [30].

**Table 4:** Postoperative outcomes of patients who did or did not suffer early recurrence.

	early recurrence (n = 35) (%)	no early recurrence (n = 101) (%)	p
Pathological T stage^a^			< 0.001
1	1 (2.9)	18 (17.8)	
2	20 (57.1)	67 (66.3)	
3	14 (40)	16 (15.9)	
4	0	0	
Pathological N stage^a^			< 0.001
0	6 (17.1)	47 (46.5)	
1	18 (51.4)	36 (35.6)	
2	11 (31.5)	16 (15.9)	
Residual tumor^a^			0.150
R0	24 (68.6)	81 (81.8)	
R1	11 (31.4)	18 (18.2)	
R2	0	0	
Adjuvant chemotherapy			0.673
Yes	26 (74.3)	76 (76.0)	
No	9 (25.7)	34 (34.0)	
Regimen of adjuvant chemotherapy			1
TS-1	13 (50.0)	37 (49.3)	
Gemcitabine	13 (50.0)	38 (50.2)	
Surgical Complications^b^			0.040
≤ Grade II	33 (94.3)	79 (79.0)	
≥ Grade III	2 (5.7)	21 (21.0)	
CR-POPF	0 (0)	16 (76.2)	
OS-SSI	1 (50)	0 (0)	
Biliary leakage	0 (0)	4 (19.0)	
Bleeding	1 (50)	1 (4.8)	

^a^ UICC TNM classification

^b^ The Clavien-Dindo Classification

CR-POPF, clinically relevant postoperative pancreatic fistula

OS-SSI, organ/space surgical site infection.

## Discussion

Distinguishing those resectable PDAC patients who will benefit from resection with curative intent prior to surgery from those who will suffer early recurrence (ER) and for whom surgery would be superfluous would be clinically useful. Here, we constructed a model comprising multiple available preoperative parameters using the Elastic Net method to more accurately predict the ER of resected PDAC. Including the whole cohort of 136 patients as the training set, the model we finally developed had a very good performance (AUC = 0.767), whose predictive power was replicated in the validation set (n = 15). Even after repeated 10-fold cross-validation using smaller numbers of patients in the training sets, the mean AUC was still 0.718. The mean AUC and net benefit in a decision curve analysis was superior to any risk factors previously reported by others or resulting from conventional multivariate analysis that we performed in parallel here. Thus, decision curve analysis indicated that the models constructed by Elastic Net could identify patients who might need alternative treatments rather than surgery to achieve good net benefit [24].

Recently, neoadjuvant chemotherapy (NAC) alone, or together with radiotherapy has become a standard treatment for borderline resectable PDAC [31–33] and several trials have investigated NAC in resectable PDAC [32, 34–36]. However, whether NAC or upfront surgery together with adjuvant chemotherapy is the better strategy remains an open question [35, 37]. It is conceivable that only a fraction of the entire resectable PDAC population is a good candidate for NAC [38], the benefits of which would be to reduce tumor size; increase the chance of later curative resection, and to control potential micrometastatic disease. On the other hand, a major disadvantage of using NAC is that there might be disease progression during chemotherapy, which would convert the tumor from resectable to unresectable in the worst case. Nevertheless, if the disease does progress during chemotherapy this may represent cases with high malignancy that might not benefit from surgery anyway [35]. In the present study, we assumed that patients experiencing recurrence within 6 months of surgery did have highly malignant cancer or already had micrometastases at the time of resection. It would be exactly these patients who might be good candidates for NAC rather than surgery. Hence, the problem is how to identify those patients at high risk of ER with sufficient accuracy to enable clinical decision-making at the level of the individual (personalize medicine).

The National Comprehensive Cancer Network guideline [39] proposes several factors as high-risk features for poor prognosis: imaging finding, very highly elevated CA 19–9, large primary tumors, large regional lymph nodes, excessive weight loss, and extreme pain. Our final model (AUC = 0.765) had better predictive power than the single factors: CA19-9 (AUC = 0.572), primary tumors’ sizes (AUC = 0.630), swelling regional lymph nodes (AUC = 0.627), and peripancreatic invasion (AUC = 0.609) by imaging studies. There are also several published reports on factors associated with early recurrence of resected PDAC, although the definition of ER was different in these studies (either 6 or 12 months). Very recently, Groot et al. reported that the Charlson age-comorbidity index, tumor size on computed tomography and CA 19–9 level were preoperative risk factors for ER [14], whereas Suzuki et al. reported that CA 19–9 and CEA were independent predictors thereof [40]. In addition, the immune-inflammatory status of the tumor microenvironment has come to the fore in recent years as playing a very important role in cancer surveillance and elimination of many cancers, including PDAC [16]. In this respect, Ikuta et al. investigated combining the CA 19–9 marker with the PLR as a predictor of early recurrence [17]. However, the mere positivity or negativity of these factors has not been a useful tool to influence in making clinical decisions. In fact, the weighting of each factor was continuous, which was a limitation of the method of analysis, especially for binomial logistic regression analysis [41]. Moreover, the situation is complex, and ER is dependent on both the grade of tumor malignancy (TNM classification, tumor markers), as well as other patient factors such as demographics and immune-inflammatory status. Therefore, as shown in the present study, the accuracy of prediction based on only one factor or a simple combination of a small number of factors is neither sufficient nor appropriate. Alternative methods need to be developed, and to the best of our knowledge, the present study is the first to use a sparse modeling method to construct a predictive model with superior performance after taking a larger number of potentially related factors into account.

Two points may be of concern for the clinical applicability of our predictive model. The first is the use of serum DUPAN2 levels. As is well known, CA 19–9 gives false-negative results in patients who are negative for the Lewis blood group phenotype, though it is the most common and investigated biomarker for pancreatic cancer [42–44]. DUPAN2 has been reported to have prognostic value as a biomarker for pancreatic cancer even in the Lewis negative blood group phenotype [43, 45, 46]. Therefore, we recommend evaluating DUPAN2 as well as CA19-9 routinely. The second is the effort required to use the equation. To make it easier to calculate the probability of early recurrence using this model, we have created an excel file and attached it as a S1 Data. We also are planning to make the excel file available for download from our department’s website.

Nonetheless, our study does have several limitations. First, measurement errors may have occurred, because the clinical evaluation of regional LN involvement and peripancreatic invasion is highly inaccurate by CT or even EUS. Although the influence of the inaccuracy does not always match, we used the same definitions for preoperative regional LN involvement and peripancreatic invasion in the present study and demonstrated that this practice had sufficient predictive power. The second is its entirely retrospective nature using data from a single institution with limited numbers of patients. To compensate for missing data, we employed a machine learning method (“factor analysis of mixed data”) and used a 10-fold cross-validation method. Even after repeating the 10-fold cross-validation 1000 times, we found that the mean AUC of our constructed models had impressive power (AUC = 0.718) relative to CA 19–9 alone (a conventional risk factor), clinical lymph node metastasis (a significant risk factor by multivariate analysis in this study), as well as the combination of CA 19–9 and PLR reported by Ikuta et al^12^, illustrating the usefulness of the method described here. However, by applying this analysis method, we expect that further model construction using data from multicenter studies with larger patient populations and external validation studies are facilitated.

## Conclusions

Patients with resectable PDAC at high risk of early recurrence may need an effective alternative (e.g. NAC) to default surgery. To identify this subgroup, we screened multiple factors to construct a predictive model with good performance, using the sparse modeling method Elastic Net. As a result, seven preoperative factors were selected: age, DUPAN2, NLR, SIII, tumor size, lymph node metastasis and is peripancreatic invasion. We believe that our method may improve prediction of early PDAC recurrence accurately enough to be used in clinical practice for personalized treatments. However, our single-center retrospective findings warrant further model construction using data from multicenter studies with larger patient populations and potentially including new biomarkers to construct a more accurate evidence-based predictive model.

## Supporting information

S1 Data (XLSX)

S1 Table (XLSX)
